# The scope of health professions education requires complementary and diverse approaches to knowledge synthesis

**DOI:** 10.1007/s40037-022-00706-y

**Published:** 2022-04-07

**Authors:** Geoffrey Norman, Jonathan Sherbino, Lara Varpio

**Affiliations:** 1 McMaster Education Research, Innovation and Theory (MERIT) program, Hamilton, Ontario, Canada; 2 Department of Medicine, McMaster University, Hamilton, Ontario Canada; 3 Center for Health Professions Education, Uniformed Services University of the Health Sciences, Bethesda, MD USA

This issue of *Perspectives on Medical Education *focuses on meta-research—the study of the research process. In the call for papers, Maggio and colleagues identified a need to better understand meta-research, as research in health professions education (HPE) expands and becomes progressively more difficult to organize, aggregate and synthesize [1]. In response to the expansion of HPE research, knowledge syntheses (e.g., literature reviews) have assumed an important role. One example is the emergence of bibliometric reviews of the field. In 2011, Rotgans identified a portion of HPE’s themes and the actors by reviewing 10,000 abstracts from six major journals [2]. One interesting finding, unrelated to content themes, was the list of the top 10 articles in the field. None of the articles were original studies. There was one systematic review and four critical reviews; the remainder were commentaries and one technical report. Similar results were found by Azer in a more recent review [3].

These analyses suggest that—at least as measured by citations—review articles and commentaries may be more highly valued than original research studies. Therein lies a paradox. The actual research that advances HPE is cited less frequently than reviews and commentaries that provide overarching summaries and interpretations of the research. The challenge then is to ensure that knowledge syntheses are designed and reported in a rigorous fashion to ensure fidelity of the summary to the source research.

Knowledge syntheses, just like research methodologies, exist on a continuum between objectivist and subjectivist orientations. Systematic reviews are part of the objectivist tradition; critical reviews are part of the subjectivist tradition. There are many other kinds of literature reviews that are scattered between these poles. This breadth and divergence of knowledge syntheses is necessary in HPE, a field that lacks the discrete borders that define the theories and methodologies of a discipline. Rather, as a field, HPE benefits from a rich diversity of philosophies that inform necessary methods. However, to harness this diversity requires that scholars adopt a similar diversity in approaches to knowledge synthesis. To illustrate, we review three forms of knowledge synthesis, each reflecting different research traditions: 1) Systematic reviews and meta-analysis, 2) Narrative reviews, and 3) Bibliometric research. We acknowledge that this list of knowledge syntheses is not comprehensive, failing to discuss scoping reviews, realist reviews and many others.

## Systematic reviews and meta-analysis

As implied by the title, there are two broad components to this domain of knowledge synthesis. The first is a systematic review, in which explicit criteria are established for keywords and databases, search strategies, and methods and criteria to select specific articles for detailed review. The second component is meta-analysis, where statistical strategies derive an overall effect size, as an appropriately weighted summation of individual study effects. This domain adheres to an objectivist orientation, where bias is mitigated and a generalizable “truth” is sought.

In HPE, unlike biomedical research, many systematic reviews are not followed by a meta-analysis. In the review cited above, only 8 of the 76 (11%) papers used meta-analysis [3]. A more recent review of the field by Maggio and colleagues identified 963 knowledge syntheses [4]. The authors reported that systematic reviews were the most common (35%). Critically, systematic reviews with meta-analysis were considered separately and accounted for 6% of the total. Thus, only 17% of systematic reviews also had a meta-analysis.

There are several explanations for the relative paucity of systematic reviews with meta-analysis in HPE, including: 1) the relative immaturity of the field, where multiple experiments interrogating a common question are lacking, preventing analysis of aggregated effect, or 2) the limited utility of meta-analyses in a field where isolation of a single variable of interest for meta-analysis is often an overly simplistic approach to problems in teaching and learning. Several authors [5–7] have taken the former stance, including Ioannidis in the present issue [5]. His critique treads a familiar course, pointing out that “*most of *[HPE’s]* literature does not meet these *[rigorous, feasible, relevant, applicable]* standards. Most papers present retrospective, uncontrolled data on single institutions and with suboptimal statistical methods*.” Ioannidis derides HPE’s literature, stating that “*experimental studies with rigorous randomized controls are a minority … The perfect study, with informative placement against any pre-existing evidence, unbiased experimental controls, long-term follow-up, and highly relevant, pragmatic outcomes may not exist*.”

The characteristics Ioannidis ascribes to the highest quality research reveal a narrow perspective on what constitutes good science. There are many examples of studies [8, 9] in HPE that use standardized interventions, randomization, valid outcomes, etc., but such studies typically focus on very short-term and one-dimensional outcomes. On the other hand, there are far more that address different kinds of questions with vastly different, but appropriate, methods. To lambaste the entire HPE field for failing to adhere to one kind of perfect study is to fail to comprehend and appreciate the many kinds of knowledge and methods needed to generate a rich and nuanced understanding of HPE. In other words, HPE needs many different kinds of knowledge to understand the art and science of educating future generations of health professionals. It is not a field for one-size-fits-all methodologies. Similarly, HPE needs many kinds of knowledge synthesis to identify the “good science” that is produced in HPE, a field where the research question determines the philosophy and subsequent methodology that will underpin a study, and where the starting point is not simply an experimental design. Therefore, to be a responsible scholar in HPE requires researchers to be ontologically, epistemologically, and methodologically well rounded [10].

Systematic reviews with meta-analysis work well when there is a sufficient pool of experimental studies with the following elements:

1. The goal of the study is to investigate an intervention;
2. The intervention is standardized;
3. The outcome is standardized; and
4. A plausible control group is available.

The fact that most systematic reviews in HPE do not incorporate meta-analysis suggests that these conditions are seldom met in practice. First, relatively few questions in education can be framed as interventions, so the standard experimental paradigm does not apply. Second, in contrast to biomedical research, HPE strongly values theory development as a strategy to facilitate generalization or transfer, and theory-based research does not easily reduce to a two group, randomized experiment. Cook and colleagues have developed a characterization of study goals and distinguish between justification (Does it work?) and clarification (Why does it work?) [11]. Meta-analysis fits well into justification frameworks; however, more and more HPE journals demand that a study question is imbedded in a theoretical framework. Finally, even for those questions that can be framed as an intervention, there are typically many variants of the specific intervention, many kinds of outcome variables, and a myriad of confounding variables in education interventions. As one example, an intervention such as problem-based learning (PBL) is not a single dose; rather, it includes a number of critical elements (e.g., problems, small groups, self-directed learning, various assessment methods, etc.), and may be implemented in different contexts (e.g., a workshop or a whole curriculum), and to different populations (e.g., preclinical, clinical, and continuing education). Outcomes may vary from learner satisfaction to performance on standardized tests to performance in practice [12]. Theory is the glue that binds these dimensions together. A question like “Does PBL work?” is effectively meaningless. As Donald Campbell, the father of experimental design in education, said:

When a researcher says that such and such an effect is true, all other things being equal, he speaks from the experience of setting a great many other things equal.

Systematic reviews with meta-analysis are a valuable approach to organizing and aggregating some of the HPE literature. However, this approach only serves a small proportion of the research. In contrast to biomedical research, there is no pyramid in HPE where the systematic review with meta-analysis sits at the top with other forms of knowledge synthesis falling lower in the hierarchy. Each kind of knowledge synthesis is complementary and, when positioned side-by-side, they collectively offer various ways to understand the complex and growing HPE literature.

## Narrative reviews

Narrative reviews adopt a subjectivist philosophy and so rely on the expertise and perspective of the authors to inform the literature synthesis. The term narrative review is an umbrella term under which many different types of reviews sit. Narrative reviews are not used by scholars who aspire to unveil the “truth” about a phenomenon; researchers engaging in narrative reviews have different aspirations. As Greenhalgh and colleagues explained in their canonical article [13], “*narrative reviews provide interpretation and critique; their key contribution is deepening understanding*.” As they elucidate, not all research questions can be answered with data (and lots of it); some questions require reflection, clarification, and insight. This is the work that a narrative review can realize.

Take, for example, the state-of-the-art literature review. The purpose of this review is to provide an account of a field’s progressive understanding of a phenomenon via a three-part argument: This is where we are now. This is how we got here. This is where we should go next. To construct this argument, researchers need to review and reflect upon the history of evolving insights into a phenomenon, to identify significant turning points in that evolution, and to propose future research directions. Not all researchers will interpret that history in the same way. Not all scholars will point to the same events as watershed moments. Nor should they. The informed wisdom of the scholars engaging in the state-of-the-art review should shape the insights developed in the review. A recent publication by Schuwirth and Van der Vleuten [14], aptly titled *A history of assessment in medical education, *serves as a powerful illustration of the contributions this type of narrative review offers to science. These two scholars—key figures in the HPE assessment research—offer a historical overview of HPE’s ever maturing thinking about learner assessment and suggest how that thinking could evolve to meet future needs and expectations. This kind of review is very difficult to craft since the authors must authentically describe in the review the underpinning evidence that they summarize (a history that typically spans decades of work) and how they drew that literature together to support the conclusions drawn. The point of this review, then, is not to offer a universal/undebatable summary of assessment. Instead, the authors offer a convincing argument, defending their interpretations, and offering insights to guide ongoing scholarship. This cannot be done by simply identifying, tabulating, and objectively summarizing publications on assessment. The state-of-the-art literature review provides insights that are rigorous, feasible, relevant, and applicable in ways that are different from systematic review. Here, different is not a judgement of quality. Instead, different is simply that—not of the same kind.

This is just one example of the unique kind of knowledge created by a narrative review. There are many other forms of narrative review, including critical, hermeneutic, and meta-ethnographic. Each type reveals phenomena in new ways. Some draw on insights or theories from other fields of inquiry (e.g., critical reviews). Some connect individual manuscripts across the whole body of literature opening new ways of interpretation (e.g., hermeneutic reviews). Others synthesize qualitative data across many publications, providing insight into the complex and nuanced range of human experience (e.g., meta-ethnographic reviews).

To reject narrative reviews because they do not adhere to a single research tradition’s objectivist roots is problematic. If we dismiss research conducted from other orientations, as Ioannidis would have us do, we stand to lose the very diversity that has made HPE an exciting field of research. Holding on to one tradition with a white-knuckled grip is not a hallmark of rigor; it is a feature of narrow thinking. HPE has a broader perspective. HPE scholars have acknowledged and made use of the power of engaging in science in many different ways.

This is not to suggest that narrative reviews are without limitations. Narrative literature reviews are informed by the researchers conducting them. This demands, then, that reflexivity strategies be included to make explicit how the authors’ stance influences the interpretation of the literature. Moreover, because search strategies used to identify relevant literature are rarely explicit, the onus is on the author to represent the literature fairly and not cite only supportive work. It is the norm that different authors will look at the same literature and draw different, sometimes very different, conclusions. It is precisely these very differences that make narrative reviews powerful tools in the HPE researcher’s toolbox.

## Bibliometric research

We discuss bibliometric meta-research only briefly, as Ninkov has written a comprehensive treatment of the subject in this issue in this issue [15].

Bibliometric research uses the same raw source material—research studies—as the other two traditions; however, the focus is very different. The primary interest appears to be using research products (e.g., publications) as a strategy to understand the nature of the scientific enterprise. The actual findings of a particular study are of no specific interest. As Ninkov states:

Bibliometrics is the analysis of published information (e.g., books, journal articles, datasets, blogs) and its related metadata (e.g., abstracts, keywords, citations) using statistics to describe or show relationships between published works.

Positioned within an objectivist philosophy, bibliometric reviews clearly add a unique perspective to our understanding of HPE. For example, in this issue, Albert and colleagues use bibliometric methods to contrast the use of disciplinary knowledge by medical education and general education researchers [16]. Rees and colleagues, in this issue, address the issue of research productivity and the tyranny of the h‑index [17]. However, bibliometric approaches neither summarize nor synthesize research findings around particular questions. This is not a flaw in design; it is the intent of the design.

Bibliometric research complements systematic reviews and narrative reviews by showing the connections and gaps between studies. It does not assist in aggregating outcomes, but instead reveals the influence of one study on another.

## Conclusion

As a field, HPE research draws on many different disciplines. This diversity is generative [18]; the interplay of methodologies informed by various research paradigms has been consistently hailed as productive and beneficial for the field of medical education. As Shulman eloquently pointed out 40 years ago [19]:

[E]ducation is a field of study, a locus containing phenomena, events, institutions, problems, persons, and processes, which themselves constitute the raw material for inquiries of many kinds. The perspectives and procedures of many disciplines can be brought to bear on the questions arising from and inherent in education as a field of study.

A consequence of this diversity is that it is not possible to devise universal standards of research quality that apply equally to all of HPE’s questions and methodologies. It makes no more sense to speak of a hierarchy of research designs in HPE research with RCT at the top and case series at the bottom than to speak of a case-control study in cosmology. There is no single, superior scientific method; the appropriate method is derived from the question being pursued. As Shulman discusses, the one commonality in the multiple research approaches addressing multiple questions in education is the notion of disciplined inquiry [19], defined by Cronbach and Suppes [20] as “[research] *conducted and reported in such a way that the argument can be painstakingly examined*.” Disciplined inquiry sets the guardrails for many different paths of inquiry that all have merit, that all have explanatory power, and that all can make significant contributions to HPE’s body of knowledge.

When it comes to research syntheses, we find one point of alignment with Ioannidis: research on research is gaining ground in many scholarly domains. However, we suggest that rather than lagging behind, HPE is powerfully engaging in this trend and has been doing so for decades. Indeed, meta-analysis was invented by educational statisticians [21], and has been used in education research for 50 years.

HPE is richer for embracing a plethora of paradigmatic orientations. This pluralistic approach to science harnesses the multidisciplinary edge effect—i.e., the generative properties that manifest in research when scholars from different academic domains collaborate [18]. To do that, however, a single approach to science (and to knowledge synthesis) cannot be positioned above all others. Where some see a chaotic maze [5], we see scholarly agility to synthesize many paths towards understanding health professions education.
